# Single-molecule nanopore sensing of proline *cis*/*trans* amide isomers†

**DOI:** 10.1039/d5sc01156f

**Published:** 2025-04-25

**Authors:** Luca Iesu, Mariam Sai, Vladimir Torbeev, Bruno Kieffer, Juan Pelta, Benjamin Cressiot

**Affiliations:** a Université Paris-Saclay, Univ Evry, CY Cergy Paris Université, CNRS, LAMBE 95000 Cergy France benjamin.cressiot@cyu.fr; b Department of Integrated Structural Biology, Institut de Génétique et de Biologie Moléculaire et Cellulaire (IGBMC), CNRS UMR 7104, INSERM U 1258, University of Strasbourg 67400 Illkirch France kieffer@igbmc.fr; c École Supérieure de Biotechnologie de Strasbourg (ESBS), CNRS UMR 7242 Biotechnology and Cellular Signalling, University of Strasbourg 67400 Illkirch France; d Université Paris-Saclay, Univ Evry, CY Cergy Paris Université, CNRS, LAMBE 91025 Evry-Courcouronnes France juan.pelta@univ-evry.fr

## Abstract

Molecules known as stereoisomers possess identical numbers and types of atoms, which are oriented differently in space. *Cis*–*trans* isomerization of proline, a distinctive case of stereoisomerism in peptides and proteins, includes the rearrangement of chemical groups around an acyl-proline amide bond that bears the partial double bond character. Many cellular processes are affected by *cis*–*trans* proline isomerization and associated conformational protein interconversions. This work explored the conformer ratio of natural and chemically modified prolines using the aerolysin pore as a nanosensor. Despite the well-known involvement of proline in protein folding, stability, and aggregation, the highly demanding discrimination of *cis* and *trans* isomers of the Xaa-Pro peptide bond has not so far been reported at a single-molecule level using an electrical detection with a nanopore. For a proline-rich 19 amino acid residue fragment of the Dynamin 2 protein, one of the subfamilies of GTP-binding proteins, the third proline in the sequence was substituted by two stereoisomeric 4-fluoroprolines. The nanopore experiments were able to sense the influence of fluorination in shifting the *cis*/*trans* conformers' equilibrium compared to the natural proline: for 4-(*R*)-fluoroproline, the *trans* amide isomer is more favored, while the opposite shift was observed for 4-(*S*)-fluoroproline. NMR spectroscopy was used to validate the nanopore results. Overall, our findings demonstrate the high sensitivity of single-molecule nanopore sensing as an analytical tool for stereoisomer identification within peptides.

## Abstract 2

Aerolysin nanopore was applied as a tool to discriminate the *cis* and *trans* isomers of the Xaa-Pro peptide bond at a single-molecule level.

## Graphical abstract

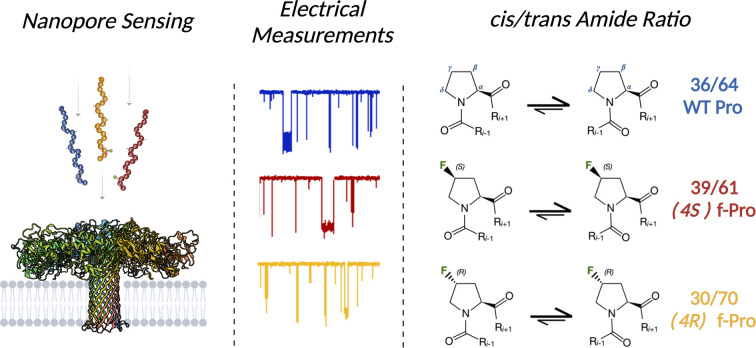

## Introduction

Spatial isomers, or stereoisomers, are molecules with the same atomic composition and chemical bond arrangement but exhibiting a different three-dimensional positioning of the atoms, resulting in distinct configurations and conformations. *Cis*–*trans* isomerization describes a specific case where isomers differ by the relative positions of chemical groups across a double bond or a ring structure. The high energy barrier between isomers generates two distinct populations of molecules with slow interconversion rates, enabling their study by physical methods (spectroscopies, X-ray diffraction, *etc.*) based on their distinct physical or chemical properties.^1^ In chemistry, well-known examples of *cis*/*trans* isomers are provided by alkene^2^ or diazene^3^ compounds for which double bonds prevent free rotation between two carbon or nitrogen atoms, respectively. As exemplified by the well-known retinal whose isomerization from 11-*cis*-retinal to all-*trans* form provides the fundamental mechanism of photon detection in many living organisms, the *cis*/*trans* isomerization has a critical role in biology.^4^ Another notable case is the isomerization of fatty acids in bacteria to control membrane fluidity.^5,6^

In proteins, the backbone conformation is defined by three dihedral angles, including the rotation around the peptide bond (so-called *ω* angle). Due to the partial double-bond nature of the peptide bond, its value is restricted to around 0 or 180°, defining the *cis* and *trans* conformations by the relative position of the neighboring C^α^ atoms either on the same side or across the peptide bond, respectively. The *cis* conformation is less energetically favorable due to the higher steric hindrance of the lateral chain groups connected to the corresponding C^α^ atoms, leading to a strongly dominant *trans* conformation for all canonical amino acids except for proline.^7^ Unlike in all other ribosomally encoded amino acids where one of the substituents at nitrogen is the smallest hydrogen, in proline, due to the pyrrolidine ring structure, the nitrogen atom is bonded to C^α^ and C^δ^, both non-hydrogen atoms. Therefore, the steric difference between *cis* and *trans* isomers is lowered.^8^ Furthermore, in proline, the delocalization of electron density within O=C–N motif and, subsequently, C–N double bond character are reduced, thus lowering the free energy barrier of *cis*–*trans* interconversion. In fact, proline *cis*–*trans* isomerization occurs spontaneously at room temperature but at relatively slow rates (timescale of seconds to hours).^9^ In most proteins, the *trans* prolyl peptide bond isomer is crucial for functionality, as shown for the triple helices constituting the collagen fibrils.^10,11^ Switching between the *trans*- and *cis*-conformers of proline profoundly impacts protein folding.^12^ This kinetics is regulated by peptidyl-prolyl isomerases (PPIases), a family of enzymes that accelerate the *cis*/*trans* isomerization rate.^13^ Malfunctioning in this kinetic regulation may result in protein misfolding and aggregation, as shown for β2-microglobulin, in which the *cis*-to-*trans* isomerization of Pro_32_ is known to trigger the misfolding, resulting in amyloid aggregation.^14–17^ The proline isomerization event is also directly involved in several molecular mechanisms, as shown for the 5-hydroxytryptamine type 3 (5-HT3) receptor, where the *cis*-to-*trans* isomerization of Pro_8_ provides the interconversion between this neurotransmitter-gated ion channel's open and closed states.^18^ Proline isomerization also plays a prominent role in cell signaling mechanisms where the *cis*–*trans* isomerization rate is modulated by the phosphorylation of the serine residue adjacent to proline, as in the Crk signaling pathway.^19^ Chemical modifications of the proline ring itself also alter its conformational properties, as shown by the hydroxylation of the C^γ^, which is highly frequent in collagen.^14^ The substitution of hydroxyl by a fluorine atom at this position has been shown to alter both *cis* and *trans* populations and the isomerization rate.^20^ This has been exploited to study the role of proline *cis*/*trans* isomerization in numerous biological systems.^21–23^

Because of these relevant roles in biology, proline isomerization has been studied by many biophysical methods, such as X-ray crystallography, Nuclear Magnetic Resonance, and, more recently, Cryo-Electron Microscopy.^24^ Due to the ability to observe molecular systems in water at room temperature and to provide kinetic measurements at time scales relevant to the *cis*–*trans* isomerization, NMR has provided many structural and kinetic insights into this phenomenon.^25–28^ The high sensitivity of the fluorine nuclear spin to its chemical environment provides novel interesting approaches where modifications of the proline's structure and dynamics upon its fluorination can be described in great detail. This has been used to study the binding kinetics of a SH3 domain to a proline-rich peptide,^29^ the solvent effect on proline *cis*/*trans* isomerization,^30,31^ or to design molecular probes for enzymatic activity.^31^

**Fig. 1:**
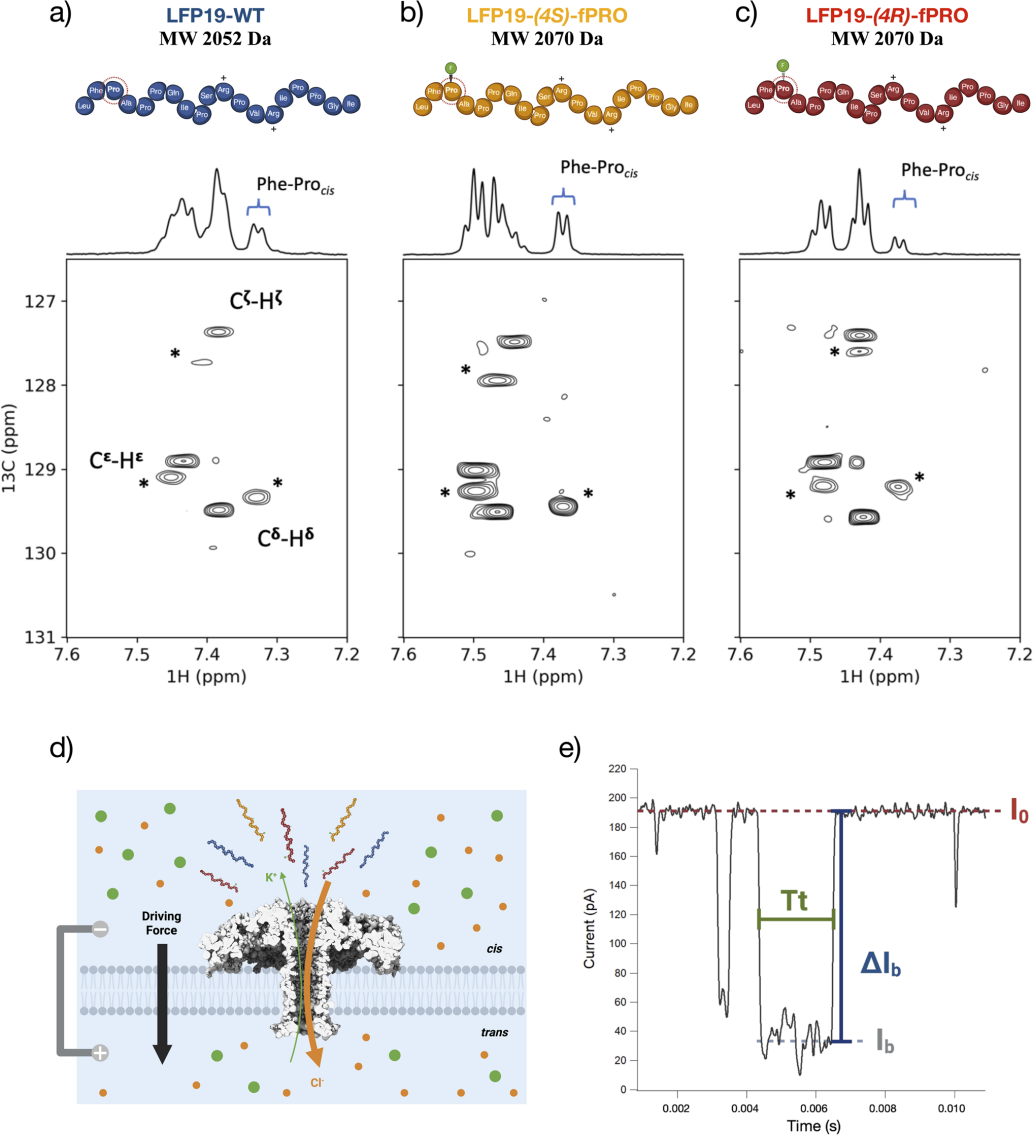
(a) ^1^H NMR spectrum and ^1^H–^13^C HSQC of the DNM2 peptides with either a natural proline (LFP19-WT), (b) a (4*S*)-fluoro-proline, or (c) a (4*R*)-fluoro-proline at position 3. The spectral region corresponds to the correlations of the aromatic carbon and protons of the preceding Phe-2 residue. The correlation peaks labeled with a star indicate the *cis* isomers of the Phe_2_–Pro_3_ peptide bond. Spectra were recorded at 313 K in D_2_O at pH 7 at 600 MHz proton frequency. (d) Schematic representation of the nanopore system used to study the peptides (in blue, LFP19-WT; in ochre, LFP19-(4*S*)-fPRO; in red, LFP19-(4*R*)-fPRO). An aerolysin protein (PDB 5JZT) is inserted in a lipid membrane (light grey) that separates two compartments filled with a buffer solution of 4 M KCl (K^+^ ions in green, Cl^−^ ions in orange). After applying a positive voltage difference, an ionic current will be present due to the ions flowing through the pore directed to the oppositely charged electrodes. (e) Example of typical blockade events showing the open pore current (*I*_0_) and the blockade current (*I*_b_), which give the blockade level (Δ*I*_b_) for a specific interval of time spent inside the pore (Tt). Image (d) created using http://Biorender.com.

Single-molecule methods provide a powerful and complementary way to study proline properties and reveal their functional role in proteins. For instance, single-molecule Fluorescence Resonance Energy Transfer (smFRET) has been used to provide quantitative information on proline *cis*/*trans* isomerization and its role in a protein involved in regulating gene expression.^32^ Nanopore sensing is another versatile single-molecule detection method whose popularity has increased in the last two decades. This technique consists of a single pore (nanometre scale) embedded in a lipidic membrane, isolating two compartments filled with an electrolyte solution^33^ (Fig. 1d). After applying an electric field, analyte molecules are driven through the pore, reducing the ionic current (Fig. 1e) for a specific blockade value (Fig. 1e) depending on the analyte size and the pore diameter.^34^ In addition, the size and charge of the analyte, its interactions with the pore, and the magnitude of the applied electrical force determine the duration of the blockade event (Fig. 1d), defining the time spent by the analyte inside the pore. Beyond its application in nucleic acid sequencing,^35–38^ this technique has been widely used to analyze and simultaneously detect peptide or protein biomarkers.^39–51^ Equally important is to identify and characterize chemical or post-translation modifications (PTMs) in proteins^52,53^ or peptides.^54–62^

The first study on nanopore sensing of molecular isomers was published in 2012 by Boersma and Bayley,^63^ and only a few numbers were published in the following years. Bayley's group distinguished the isomers of d-glucose and d-fructose using an alpha-hemolysin engineered with boronic acid.^64^ In contrast, Long's group used an aerolysin nanopore to identify single oligonucleotide *cis*/*trans* photoisomers accurately.^65^ Huang's group used machine learning and *Mycobacterium smegmatis* porin A, modified with a phenylboronic acid adapter. They discriminated the catecholamine's enantiomers (chiral isomers)^66^ and the fifteen alditol epimers (diastereoisomers) in sugar-free drinks and healthcare products.^67^ Furthermore, they discriminated disaccharide isomers of sucrose, lactose, and maltose,^68^ and, the *cis*-diols in fruits.^69^ Recently, Long's group achieved the identification of tetrasaccharides differing in only one glycosidic bond by using the electrostatically asymmetric OmpF and machine learning.^70^ Speaking of peptides, the OmpF nanopore has been used to distinguish between chiral amino acids and positional isomers^71^ and isomeric PTMs using an aerolysin mutant.^72^ Also, interesting work was published by Maglia's group in discerning constitutional isomers, enantiomers, and diastereoisomers using wild-type and mutant CytK and FraC nanopores.^73^ Finally, Pelta's group used machine learning and aerolysin to identify and detect the vasopressin biomarker and its enantiomer.^74^

Besides classical methods used in structural biology, studying *cis*/*trans* conformational and configurational isomers using nanopores is appealing to reduce costs and provide access to higher throughputs. However, the challenging discrimination between *cis* and *trans* isomers of the Xaa-Pro peptide bond with nanopores has never been undertaken so far. To address this, we have investigated a 19-residue peptide from the DNM2 protein spanning the proline-rich region from residues 826 to 845. Mutations within the *DNM2* gene of this protein cause centronuclear myopathies (CNM)^75^ by disrupting its interaction with BIN1, a protein that contains an SH3 domain specialized in proline-rich peptide recognition. ^1^H NMR investigations of this peptide revealed that the first proline at position 3 has a *cis* population significantly larger than the six remaining prolines within the peptide sequence (Fig. 1a–c). At the single-molecule level using nanopore, we could detect the predicted *cis*–*trans* conformational equilibrium for Pro_3_ with population ratios comparable to those found by NMR. Thanks to well-defined experimental settings, we also determined the entry orientation of peptides inside the sensing area of aerolysin.

## Results and discussion

### Aerolysin nanopore sensing

The approach was benchmarked using a peptide of 19 amino acids spanning a proline-rich region of the DNM2 protein. This peptide (abbreviated as “LFP19-WT”) harbors seven proline residues, and 2D NMR spectra revealed the presence of a large conformational heterogeneity due to proline *cis*/*trans* equilibrium. Assignment of ^1^H and ^13^C resonances and the substitution of the first proline (Pro_3_) by the 4-(*R*)- and 4-(*S*)-fluorinated proline analogues resulting in LFP19-(4*S*)-fPRO and LFP19-(4*R*)-fPRO peptides allowed the identification of the Phe_2_–Pro_3_ peptide bond as one of the sources of this heterogeneity with a significant population of the *cis* isomer. The presence of *cis* and *trans* isomers induces two distinct sets of resonances for aromatic resonances of Phe_2,_ enabling a semi-quantitative estimate of their respective populations (Fig. 1). For LFP19-WT, the population of the Phe_2_–Pro_3_ peptide bond *cis* isomer is estimated to be 34%, according to the respective integrals of Phe_2_ Cδ–Hδ and Cε–Hε correlation peaks assigned to the *trans* and *cis* conformations (indicated by a star in Fig. 1b). Substituting Pro_3_ with the 4-(*S*)-fluoroproline leads to an increase of up to 43% in the *cis*-amide isomer population. In comparison, inserting 4-(*R*)-fluoroproline at this position reduces the population of the *cis* isomer down to 22%. These observations are consistent with the known stereo-electronic effect of the fluorine atom at position Cγ (or C4), shifting the equilibrium either to *cis* or to *trans* isomers for the 4-(*S*)- and 4-(*R*)-fluoroprolines, respectively. Noteworthy, the population of the *cis* isomer (34%) observed for the unmodified peptide (LFP19-WT) is significantly larger than expected from statistical analysis of 3D structures reporting less than 5% of *cis* isomer. This is attributed to the interaction between the aromatic ring of the phenylalanine and the CH_2_ groups of the proline side chain that have been reported to stabilize the proline *cis* isomer.^76^

To study these peptides using a single molecule approach, we used a powerful sensor, an aerolysin wild-type nanopore^77–80^ (Fig. 1d), which is characterized by a unique geometric structure consisting of a narrow (∼1.0 nm) and long-barrel channel (∼10 nm). This structure defines a super-confined environment for single-molecule sensing. Electrical measurements under different voltage conditions allowed us to obtain raw current traces from which different parameters (Fig. 1e) were extracted to elucidate the *cis*/*trans* conformational preference for each proline and understand the transport dynamics.

**Fig. 2:**
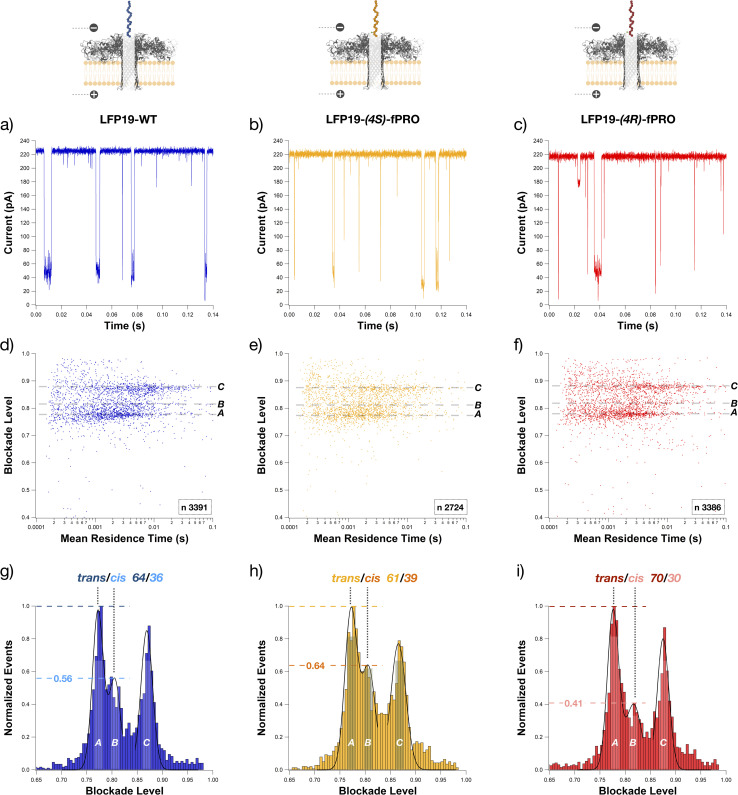
Electrophysiological results of the independent measurements of each peptide (in blue, LFP19-WT at 15 μM – (a, d and g); in ochre, LFP19-(4*S*)-fPRO at 15 μM – (b, e and h); in red, LFP19-(4*R*)-fPRO at 10 μM – (c, f and i)) under +100 mV in 4 M KCl and 25 mM Tris – pH 7.5 using an aerolysin nanopore. Examples of open pore current and blockade events occurring when the peptide blocks the pore are shown in the typical current traces *versus* time filtered at 5 kHz. (d–f) Scatter plots of normalized average blockade level (Δ*I*_b_) against the mean residence time (Tt); three main populations of points are appreciable *A*, *B*, and *C*, ranging from 0.7 to 0.9 of blockade level. (g–i) Histograms of the normalized average blockade level (Δ*I*_b_) against the normalized number of events fitted with a Bi-Gaussian followed by a LogNormal (black lines). These fittings were used to obtain each population's most probable blockade level values (shown in the figure). The values above the dashed lines correspond to the height of the second population in terms of the normalized number of events. Experiments were reproduced two to three times for each peptide. *N* corresponds to the number of events.

Due to almost equivalent *cis* and *trans* populations, the LFP19-(4*S*)-fPRO peptide was used to determine the effect of salt on the electrical sensing at +100 mV. Compared to the open pore current in 4 M KCl (Fig. 2a–c), we observed a decrease in 4 M LiCl, while the measured value in 4 M CsCl was also lower (ESI Fig. S1†). Interestingly, for 4 M LiCl, a lower frequency of interactions between the analyte and the nanopore is appreciable by noting the low frequency of blockade events in the current trace for the same timescale. This means that the solvation of cation has a significant effect on the driving force, as has already been reported in the literature.^81^ For the same reason, these two salts gave us different blockade level values and mean residence times. Notably, in 4 M LiCl, we obtained one single dominant population, whereas in 4 M CsCl three populations were detected as in 4 M KCl. Due to the higher frequency of events at the same peptide concentration (ESI Fig. S2 and Table S1†), we opted to continue the experiments in 4 M KCl. Additionally, we only observe three population of events from +100 mV in 4 M CsCl, limiting for a robust statistical analysis (ESI Fig. S3†). To show that 4 M KCl is the best salt concentration, we performed measurements at 1 M KCl (ESI Fig. S4†). The data show that it is not possible to discern the three event populations anymore, even at higher peptide concentrations.

In the current traces under +100 mV in 4 M KCl, deep blockade events are shown for each peptide in a selected interval of 0.14 s (Fig. 2a–c). The events indicate that the peptides are entering the nanopore. Each current trace analysis leads to a set of points, thanks to which a statistical analysis of the blockade level was performed (Fig. 2d–f). The scatter plots allow for the distinguishing of three main populations, *A*, *B*, and *C*, with a partial overlap of *A* and *B*, both being very distinct from *C* (values reported in Fig. 2g–i and ESI S5;† examples of electrical blockade events in ESI Fig. S6–S8†). This observation led us to search for a trend in the points extrapolated for *A* and *B*, following the hypothesis that they could correspond to the two global conformations induced by the isomerization of Pro_3_.

### Identification of *trans*–*cis* proline equilibrium

We demonstrated that the aerolysin nanopore could sense the *cis*/*trans* conformer of natural and chemically modified proline by discerning between the two induced global conformational changes. Firstly, in the histogram from data under + 100 mV (two to three replicates), we observed that population B's normalized by number of events, overlapping with population *A*, is changing among the three peptides (Fig. 2g–i). This trend is repeated at different voltage conditions (two to three replicates for each voltage; ESI Fig. S5†), and the height of population *B* stays in a different interval for each peptide (ESI Fig. S5 and Table S2†). Based on the opposite conformational bias introduced by the fluorinated prolines, either shifting the equilibrium towards *cis* isomer of the Phe_2_–(4*S*)-fPro_3_ peptide bond in the LFP19-(4*S*)-fPRO peptide or *trans* isomer in the LFP19-(4*R*)-fPRO peptide, we compared the ratio between population *A* and population *B* for these two peptides.

**Fig. 3:**
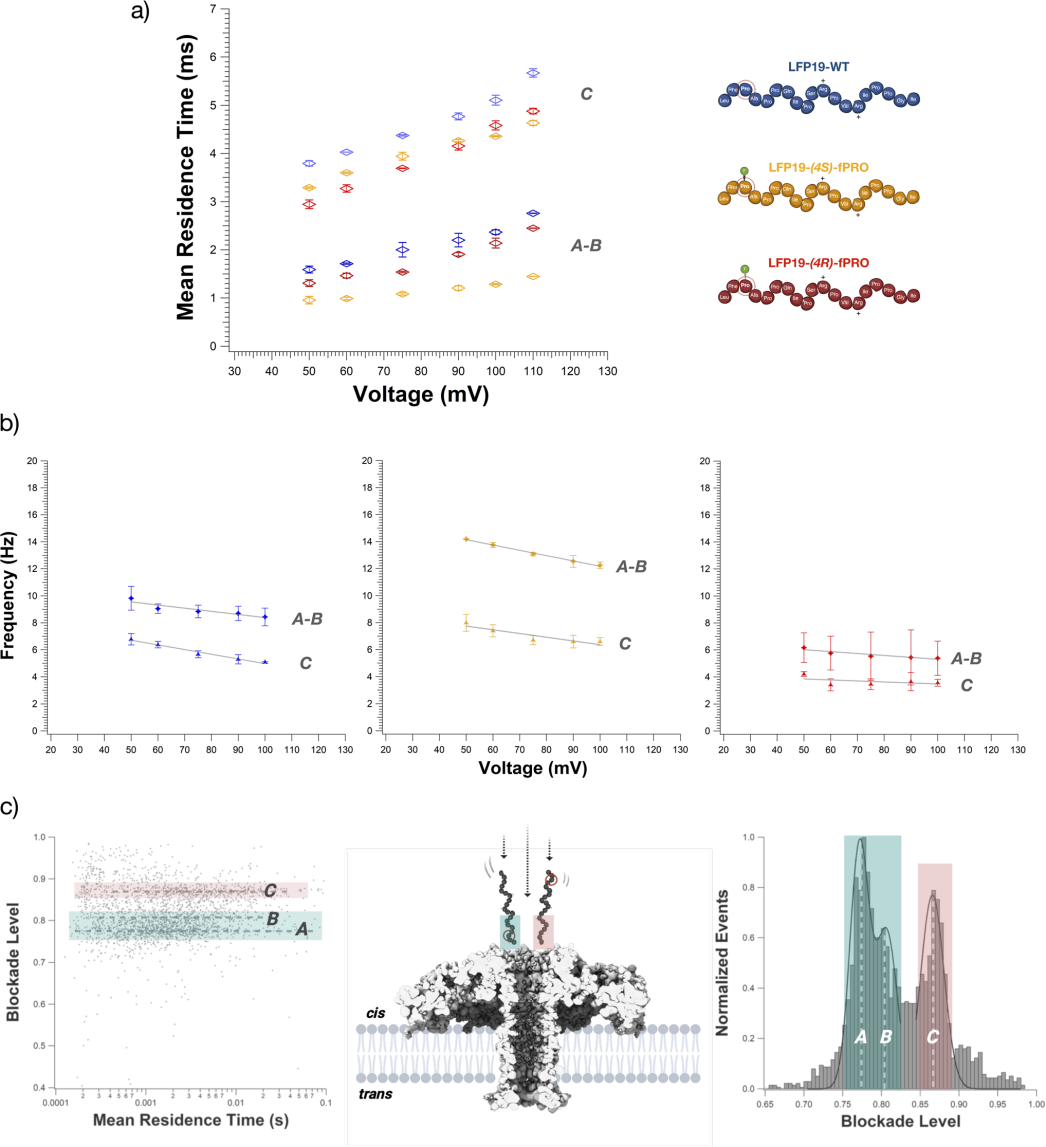
Transport dynamics and entry orientation. (a) Increase of the mean residence time for each peptide as a function of the voltage applied. In blue, markers for LFP19-WT; in ochre, markers for LFP19-(4*S*)-fPRO; in red, markers for LFP19-(4*R*)-fPRO. *A* and *B* populations have been considered single populations for the mean residence time values due to their overlapping condition. Markers and error bars correspond to the mean residence time and standard deviation; these values come from three individual fits of 2 or 3 independent experiments. (b) Different interaction frequencies of peptides' entry orientations into the aerolysin nanopore. Each peptide has been measured independently in 4 M KCl, 25 mM Tris – pH 7.5; peptide concentration: 15 μM. Points and error bars represent the mean and the standard deviation of frequency values calculated from 3 fits from 2 independent recordings. Frequency values refer to events selected according to the following criteria: blockade duration from 200 μs to 1 s; blockade amplitude depending on the voltage; noise from 1 pA to 100 pA. (c) The three main populations are highlighted in the scatter plot and the histogram. Events of *A* and *B* populations correspond to the peptide entering the aerolysin nanopore *via* its N-terminus; events of population *C* correspond to the peptide entering the aerolysin nanopore *via* its C-terminus.

The relative fraction of population A to the sum of A and B populations was compared among the three peptides. The formula is *a*/(*a* + *b*) where *a* is equal to 1 (the highest value for normalized events) and corresponds to the height of population *A*. *b* is equal to the height of population *B*, therefore is changing among the three peptides (Fig. 2g–i; ESI Fig. S5 and Table S2†). Under + 100 mV, the *trans*/*cis* ratio is 64/36 for LFP19-WT, 61/39 for LFP19-(4*S*)-fPRO, and 71/29 for LFP19-(4*R*)-fPRO (Fig. 2g–i and ESI Table S2†). Notably, these results are in good agreement with the corresponding *trans*/*cis* ratios determined from NMR data. The height of population *B* under different voltage conditions is indicated for each peptide in ESI Fig. S5 and Table S2.† The blockade level values for each peptide are almost equal under the same voltage applied (ESI Fig. S5†). Therefore, it was impossible to discern among them using the blockade level values as a discriminant in these experimental conditions. Nevertheless, we can distinguish them thanks to their different mean residence times (except under +90 mV due to the overlapping of the error bar in Fig. 3a) and their different interaction frequencies with the nanopore (Fig. 3b).

### Transport dynamics and entry orientation

The main driving force responsible for the peptides' entering the nanopore is the electroosmotic flux (EOF) from the *cis* to the *trans* side of the bilayer membrane (Fig. 1d and ESI S9†). Applying a positive voltage, the slightly positively charged peptides still induce blockade current events despite the antagonistic electrophoretic force (EPF) from the *trans* to *cis* side. Under negative applied voltages, the EOF drives the peptides away from the pore, but the EPF is still strong enough to observe transient blockade events (ESI Fig. S10†). Unfortunately, these blockade events are non-resolutive.

Regarding the transport dynamics, it is possible to confirm the absence of peptide translocation due to the increase of the mean residence time (ESI Fig. S11–S13†) as the voltage applied increases for all three peptides and their corresponding populations *A*–*B*, and *C* (Fig. 3a).

Given the *trans*/*cis* ratio found between the overlapping populations *A* and *B* (Fig. 3c), we attributed these two signals to the peptide entering the nanopore *via* its N-terminus, where the proline or 4-fluoroprolines expected to undergo isomerization are positioned. In contrast, according to the presence of a single population, population *C* corresponds to the peptide entering the nanopore by its C-terminus (Fig. 3c). Therefore, these two different entry orientations can be responsible for two different timings of the peptide within the aerolysin. If the peptide enters *via* its N-terminus, it will spend less time, while the opposite happens if it enters *via* its C-terminus side (Fig. 3a). The difference in the mean residence time among all three peptides can be related to the fluorination of Pro_3_. Indeed, the peptide LFP19-WT spends more time inside the nanopore, entering *via* N-terminus or C-terminus. On the contrary, due to the fluorination and its consequent increase of hydrophobicity^82–84^ and moiety volume,^85^ the peptides LFP19-(4*R*)-fPRO and LFP19-(4*S*)-fPRO spend less time inside the nanopore. Previously, similar structure and conformation-related effects on mean residence time were reported.^50^

## Conclusion

This paper demonstrates the aerolysin nanopore's ability to detect the interconversion of *cis*/*trans* conformational amide isomers of natural and chemically modified prolines at the single-molecule level. We defined the entry orientations of these peptides thanks to the unique features of the events related to the induced conformational change at the N-terminus by the *cis*/*trans* isomerization. We show that the analysis of blockade levels discriminates three populations, two of which share the same entry orientation but differ in the *cis* and *trans* populations of a single peptide bond within a proline-rich peptide. Multiple populations were already observed in single peptide detection by nanopore, and they are usually attributed to different conformations^51^ or two different entry orientations. Again, nanopore sensitivity has been pushed forward, defining electrical signals corresponding to molecular features, such as the three-dimensional arrangement of atoms in a molecule. Given the precision of our three different *cis*/*trans* ratios, aerolysin is a suitable candidate for detecting proline *cis*/*trans* conformational states and can be then used to study other relevant proline residues. Another application could be the evaluation of different inhibitors of PPIases by comparing the proline conformational ratio measured before and after the inhibition. Considering the overexpression of PPIases in most cancers and the difficulties in measuring the *cis*/*trans* ratio, nanopore would be an efficient and direct method to screen the inhibitors. In so doing, the application of nanopore sensing in the biomedical field can be further extended.

## Data availability

The data supporting this article have been included as part of the ESI.†

## Author contributions

L. I. performed the nanopore experiments and wrote the first draft of the manuscript. M. S., B. K., and V. T. provided peptides for the study. J. P. and B. C. designed the nanopore experiments. L. I., V. T., B. K., J. P., and B. C. participated in editing the manuscript.

## Conflicts of interest

There are no conflicts to declare.

## Supplementary Material

SC-OLF-D5SC01156F-s001
