# Supplementary material for: Single-molecule nanopore sensing of proline cis/trans amide isomers
Source: Chem Sci. 2025 Apr 25;16(22):9730–8. doi: 10.1039/d5sc01156f (PMC12045290; doi:10.1039/d5sc01156f)
Supplement: SC-016-D5SC01156F-s001 [file SC-016-D5SC01156F-s001.pdf]

Supplementary Information (SI) for Chemical Science.
This journal is © The Royal Society of Chemistry 2025

Supporting Information

Single-Molecule Nanopore Sensing of *cis/trans* Conformers from Natural and Chemically Modified Proline

Luca Iesu[a], Mariam Sai[b], Vladimir Torbeev[c], Bruno Kieffer[b*], Juan Pelta[d*] and Benjamin Cressiot[a*]

[a] Université Paris-Saclay, Univ Evry, CY Cergy Paris Université, CNRS, LAMBE, 95000, Cergy, France
[b] Department of Integrated Structural Biology, Institut de Génétique et de Biologie Moléculaire et Cellulaire (IGBMC), CNRS UMR 7104, INSERM U 1258, University of Strasbourg 67400 Illkirch (France)
[c] École Supérieure de Biotechnologie de Strasbourg (ESBS), CNRS UMR 7242 Biotechnology and Cellular Signalling, University of Strasbourg 67400 Illkirch (France)
[d] Université Paris-Saclay, Univ Evry, CY Cergy Paris Université, CNRS, LAMBE,91025 Evry- Courcouronnes, France

*corresponding authors: kieffer@igbmc.fr, juan.pelta@univ-evry.fr, benjamin.cressiot@cyu.fr

Table of Contents

Methods	
Figure S1	Measurements of 15 µM LFP19-*(4S)*-fPRO at +100 mV testing different salts to study the *cis/trans* ratio of proline: 4M LiCl and 4M CsCl
Figure S2	Fittings used to determine the global frequency of 15 µM LFP19-*(4S)*-fPRO at +100 mV
Figure S3	Measurements of 15 µM LFP19-*(4S)*-fPRO under different voltage conditions in 4M CsCl
Figure S4	Measurements of 30 µM LFP19-*(4S)*-fPRO at +100 mV in 1M KCl
Figure S5	Different voltage conditions to study the *trans*/*cis* ratio of natural and chemically modified proline
Figure S6	Randomly picked up events of LFP19-WT
Figure S7	Randomly picked up events of LFP19-*(4S)*-fPRO
Figure S8	Randomly picked up events of LFP19-*(4R)*-fPRO
Figure S9	Direction of the driving forces
Figure S10	Peptide LFP19-WT under negative voltages in 4M KCl
Figure S11	Fittings used to determine the mean residence time for LFP19-WT
Figure S12	Fittings used to determine the mean residence time for LFP19-*(4S)*-fPRO
Figure S13	Fittings used to determine the mean residence time for LFP19-*(4R)*-fPRO
Table S1	Global frequency values of 15 µM LFP19-*(4S)*-fPRO 4M KCl and 4M CsCl.
Table S2	Values of single voltage average and the total average of *trans/cis* ratio

Methods

Peptide Sequences

LFP19-WT	L F P A P P Q I P S R P V R I P P G I
LFP19-*(4S)*-fPRO	L F (*4R*-fP) A P P Q I P S R P V R I P P G I
LFP19-*(4R)*-fPRO	L F (*4S*-fP) A P P Q I P S R P V R I P P G I

Peptide synthesis

The peptide LFPAPPQIPSRPVRIPPGI ("LFP19-WT") was synthesized by Fmoc/tBu-strategy on a Liberty Blue (CEM Corporation, Matthews, USA) microwave automatic synthesizer using a Rink Amide resin (100-200 mesh, loading 0.14 g/mol) on a 0.1 mmol scale. To perform coupling reactions, the coupling reagent DIC (1 mL, 0.5 M), the activator Oxyma Pure (0.5 mL, 1 M) and the amino acid (2.5 mL, 0.2 M) were added to the resin and the suspension was treated at 90 °C for 2 min (170 W for 15 s, 30 W for 110 s). Fmoc-*L*-amino acids and resins were purchased from Iris Biotech (Marktredwitz, Germany). 4-(*R*)- and 4-(*S*)-fluoroprolines were purchased from Bachem (Bubendorf, Switzerland). The Fmoc-protection was carried in house according to published protocols. Fmoc-protected fluoroprolines were purified by column chromatography (DCM/MeOH, 98/2 v/v) and the purity was checked by ^{1}H NMR.

NMR experiments

All peptides were dissolved in a freshly prepared Na-phosphate buffer (50 mM) at pH 7.2 in H_2O, then lyophilized and re-dissolved in D_2O. The concentration of the samples varied was 1.5 mM (measured by NanoDrop at 205 nm). 1D ^{1}H spectra and ^{1}H-^{13}C HSQC were acquired on a 600 MHz (^{1}H frequency) Bruker Avance I spectrometer equipped with a cryogenic QCI-F probe. The sample was packaged in a 3mm tube and the temperature was set to 313K. For ^{1}H-^{13}C HSQCs, parameters were set to specifically observe the aromatic correlations. The carrier frequencies were set to 7.4 and 130 ppm for the proton and the carbon channels, respectively. The size of the acquisition matrix was 1024 x 200 points with corresponding spectral widths of 4 and 10 ppm for the proton and the carbon dimensions, respectively. The number of scans was set to 64 with an interscan delay of 2 s leading to total acquisition time of 8 hours. Quadrature detection in the indirect dimension (F1) was performed using pulse field gradients selection of echo and anti-echo coherences (pulse program: hsqcetgpsp). The spectra were processed using Topspin 3.6 (Bruker BioSpin). Fourier transform was performed after application of gaussian and square sinebell windows on time domain signals leading to a 2048(^{1}H) x 1024(^{13}C) correlation spectrum. The assignments and peak integration were done using CcpNmr V3.[1]

Aerolysin Nanopore Production and Activation

Wild-type proaerolysin was produced by Dreampore S.A.S. (Cergy, France) as described earlier[2]. In brief, after periplasmic expression of C-terminally his-tagged protein in *E. coli* BL21-Rosetta2 (MilliporeSigma) cells were disrupted by osmotic shock to harvest the proteins. Then, two steps of purification, nickel affinity and buffer exchange chromatography (Cytiva,

Malborough MA, USA), were executed to obtain a final concentration of ~1.7 μM proaerolisin stored in 350 mM NaCl at 4 °C until next use. Before use, proaerolysin monomers were activated using trypsin immobilized on agarose beads (Thermo Scientific, Waltham MA, USA) for 15 min at RT.

Electrophysiology Measurements

Resistive pulse experiments were performed by using a vertical planar lipid bilayer setup (Warner Instruments, Hamden CT, USA). As previously described[3], a 1% (w/v) stock solution of DPhPC/*n*-decane was used to paint in a 150 μm aperture a lipid membrane separating two compartments filled with 1 mL of electrolyte solution. The electrolyte solutions were different in counterions used: 25 mM Tris, pH 7.5 in 4M KCl (majority of experiments) or in 4M LiCl, or in 4M CsCl (tests). Once achieving a single aerolysin nanopore insertion, LFP9-WT or LFP-3Pro-4R, or LFP-3Pro-4S, were added at different concentrations (10 μM, or 15 μM especially for frequency) to the *cis* and *trans* compartment. A difference of potential was applied through Ag/AgCl electrodes to measure the current signal by using an Axopatch 200B amplifier (Molecular Devices, LLC, San Jose CA, USA). Data were recorded at 250 kHz intervals (4 μs sampling time) and filtered at 5 kHz using a DigiData 1440A digitizer (Molecular Devices, LLC, San Jose CA, USA) and and a Clampex software (Axon Instruments, Union City, CA, USA).

Nanopore Data Analysis

Data processing, analysis, and plot generation were conducted with Igor Pro Software (Wavemetrics Inc., Portland, OR, USA) by using in-house alghoritms. The open pore current (I_0) and its standard deviation (σ) were used in the calculation $I_0 - 7\sigma$ to statistically determine a threshold to define blockade events corresponding to the sensing of peptides analysed[4] (Figure 1 d, Figure 2 a, b, c). Each blockade events was charachterized by multiple parameters on the base of the open pore current (I_0) and the average current blockade level (I_b) within the event and its length time (Tt). The I_b was used to calculate the normalized blockade level: $(I_0 - I_b)/I_b$. In case of low frequency events (i. e. Figure S1, S3) extracted parameters were concatenated to increase the realiability of statistics.

Supplementary References

(1) Vranken, W. F.; Boucher, W.; Stevens, T. J.; Fogh, R. H.; Pajon, A.; Llinas, M.; Ulrich, E. L.; Markley, J. L.; Ionides, J.; Laue, E. D. The CCPN Data Model for NMR Spectroscopy: Development of a Software Pipeline. *Proteins* **2005**, *59* (4), 687–696. https://doi.org/10.1002/prot.20449.

(2) Iacovache, I.; Degiacomi, M. T.; Pernot, L.; Ho, S.; Schiltz, M.; Dal Peraro, M.; Van Der Goot, F. G. Dual Chaperone Role of the C-Terminal Propeptide in Folding and Oligomerization of the Pore-Forming Toxin Aerolysin. *PLoS Pathog* **2011**, *7* (7), e1002135. https://doi.org/10.1371/journal.ppat.1002135.

(3) Cressiot, B.; Braselmann, E.; Oukhaled, A.; Elcock, A. H.; Pelta, J.; Clark, P. L. Dynamics and Energy Contributions for Transport of Unfolded Pertactin through a Protein Nanopore. *ACS Nano* **2015**, *9* (9), 9050–9061. https://doi.org/10.1021/acsnano.5b03053.

(4) Oukhaled, A.; Bacri, L.; Pastoriza-Gallego, M.; Betton, J.-M.; Pelta, J. Sensing Proteins through Nanopores: Fundamental to Applications. *ACS Chem. Biol.* **2012**, *7* (12), 1935–1949. https://doi.org/10.1021/cb300449t.

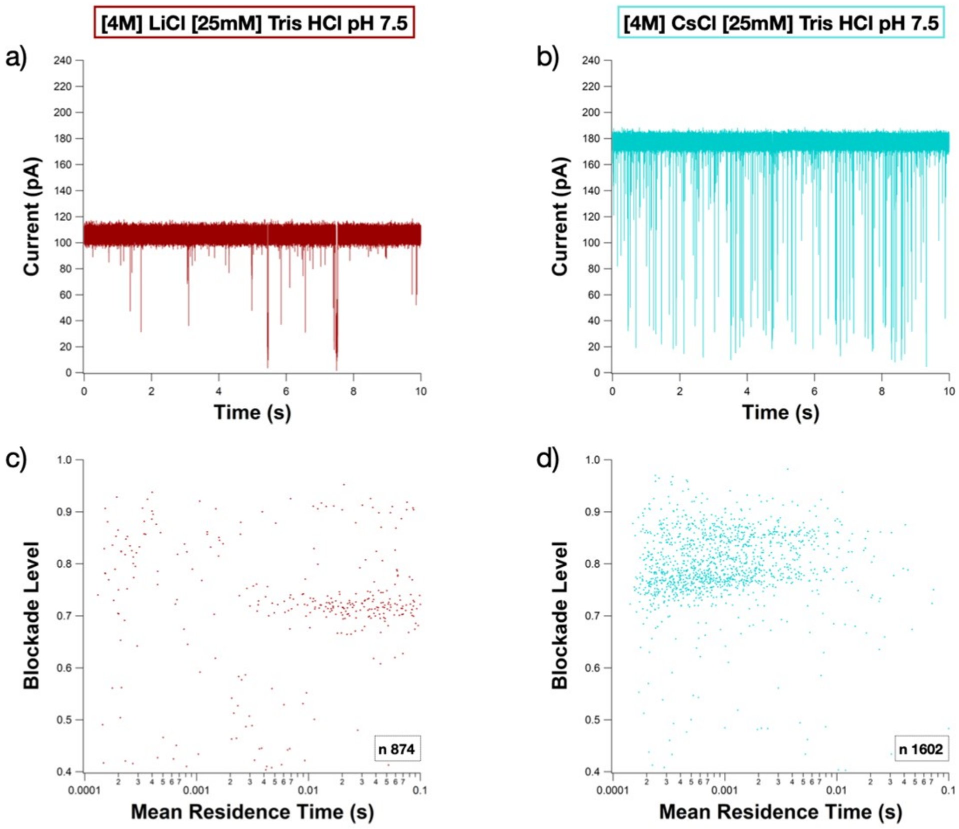

Figure S1. Measurements of 15 μM LFP19-*(4S)*-fPRO at +100 mV testing different salts to study the *cis/trans* ratio of proline: 4M LiCl in carmine red (a, c), and 4M CsCl in light cyan (b, d). (a, b) 10 seconds of current traces show lower open pore current and fewer events in LiCl despite of CsCl. (c, d) Scatter plots of normalized average blockade level (ΔI_b) against the mean residence time (Tt). In c) a single population is present between 0.65 and 0.75 of blockade level, instead, in d) multiple populations of points are appreciable, ranging from 0.7 to 0.9 of blockade level. Data analysed from n_{LiCl} = 874 events per 7 min of recorded trace; n_{CsCl} = 1602 events per 3.5 min of recorded trace.

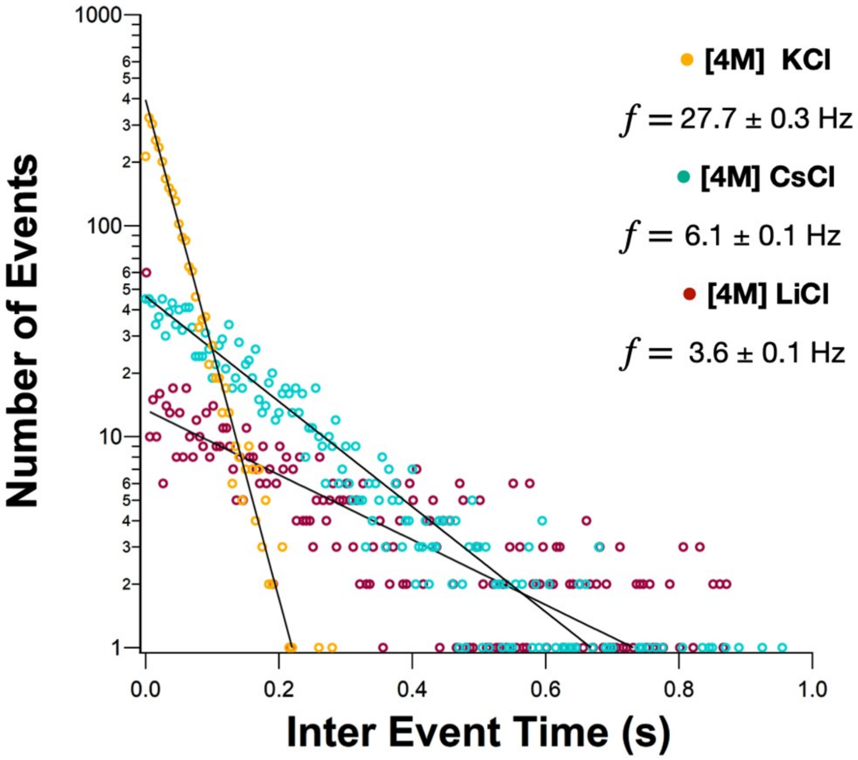

Figure S2. Fittings used to determine the mean global frequency of 15 µM LFP19-*(4S)*-fPRO at +100 mV. In 4M KCl (ochre), 4M CsCl (cyan), and 4M LiCl (carmine red).

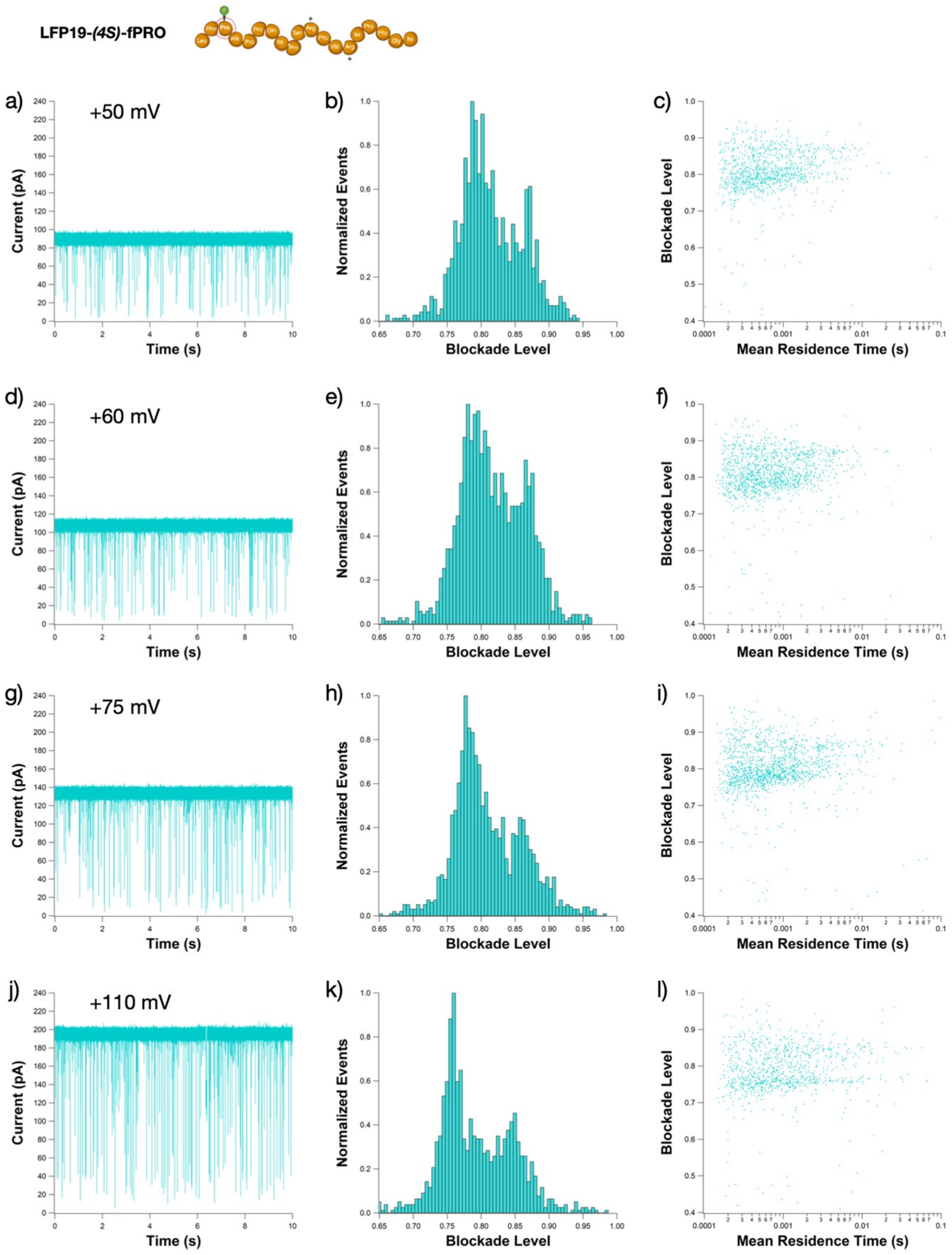

Figure S3. Measurements of 15 μM LFP19-*(4S)*-fPRO under different voltage conditions in 4M CsCl. (a, d, g, j) current traces at different voltages for a timescale of 10s. (b, e, h, k) histograms of the normalized number of events against the normalized average blockade level (ΔI_b); no fitting due to the heterogeneous distribution of points. (c, f, I, l) Scatter plots of normalized average blockade level (ΔI_b) against the mean residence time (Tt).

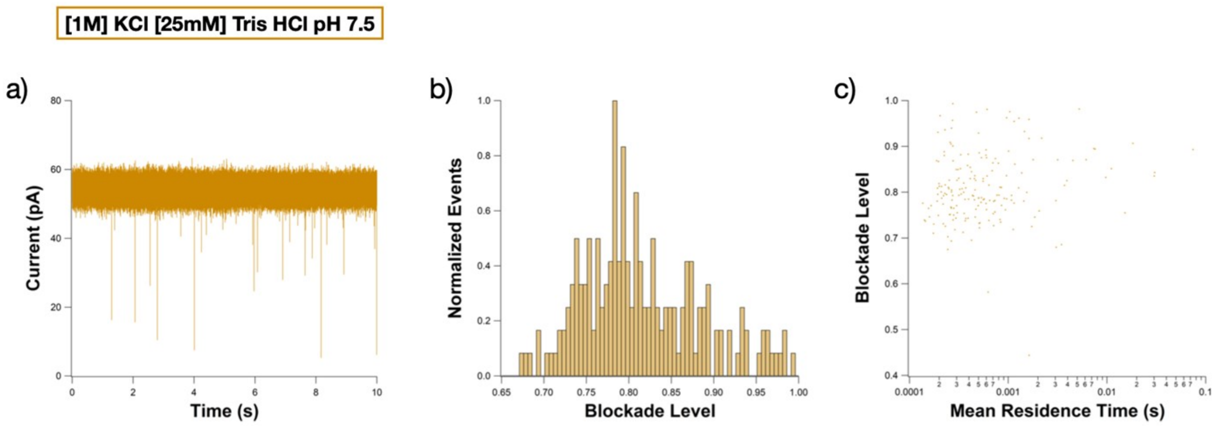

Figure S4. Measurements of 30 µM LFP19-*(4S)*-fPRO at +100 mV in 1M KCl. a) current trace for a timescale of 10s; b) histogram of the normalized number of events against the normalized average blockade level (ΔI_b); c) scatter plot of normalized average blockade level (ΔI_b) against the mean residence time (Tt).

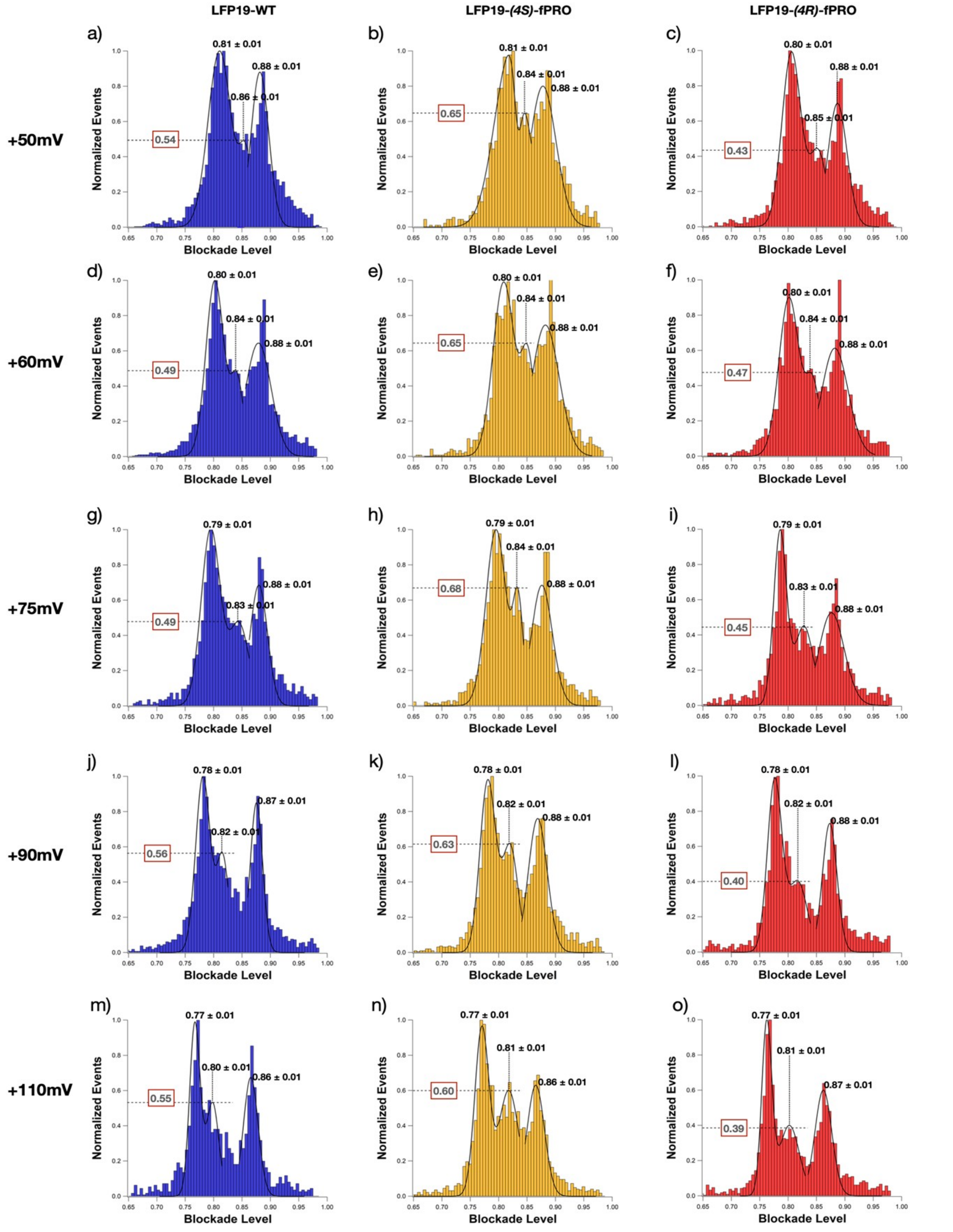

Figure S5. Different voltage conditions to study the *trans*/*cis* ratio of natural and chemically modified proline. Histograms of the normalized average blockade level (ΔI_b) against the normalized number of events: in blue, LFP19-WT; in ochre LFP19-*(4S)*-fPRO; in red LFP19-*(4R)*-fPRO. Events under +50 mV: a) $n_{LFP19\text{-}WT}$ = 2733; b) $n_{LFP19\text{-}(4S)\text{-}fPRO}$ = 3107; c) $n_{LFP19\text{-}(4R)\text{-}fPRO}$ = 3145. Events under +60 mV: d) $n_{LFP19\text{-}WT}$ = 5306; e) $n_{LFP19\text{-}(4S)\text{-}fPRO}$ = 2475; f) $n_{LFP19\text{-}(4R)\text{-}fPRO}$ = 2184. Events under +75 mV: g) $n_{LFP19\text{-}WT}$ = 2753; h) $n_{LFP19\text{-}(4S)\text{-}fPRO}$ = 2651; i) $n_{LFP19\text{-}(4R)\text{-}fPRO}$ = 2768. Events under +90 mV: j) $n_{LFP19\text{-}WT}$ = 5190; k) $n_{LFP19\text{-}(4S)\text{-}fPRO}$ = 3582; l) $n_{LFP19\text{-}(4R)\text{-}fPRO}$ = 2854. Events under +110 mV: m) $n_{LFP19\text{-}WT}$ = 1855; n) $n_{LFP19\text{-}(4S)\text{-}fPRO}$ = 3737; o) $n_{LFP19\text{-}(4R)\text{-}fPRO}$ = 4147. Black lines correspond to the Bi-Gaussian and to the LogNormal fittings applied to obtain the most probable blockade level values (shown in the figure) for each population. Experiments were reproduced two to three times for each peptide. The values inside the red box correspond to the height, in terms of normalized number of events, of the second population.

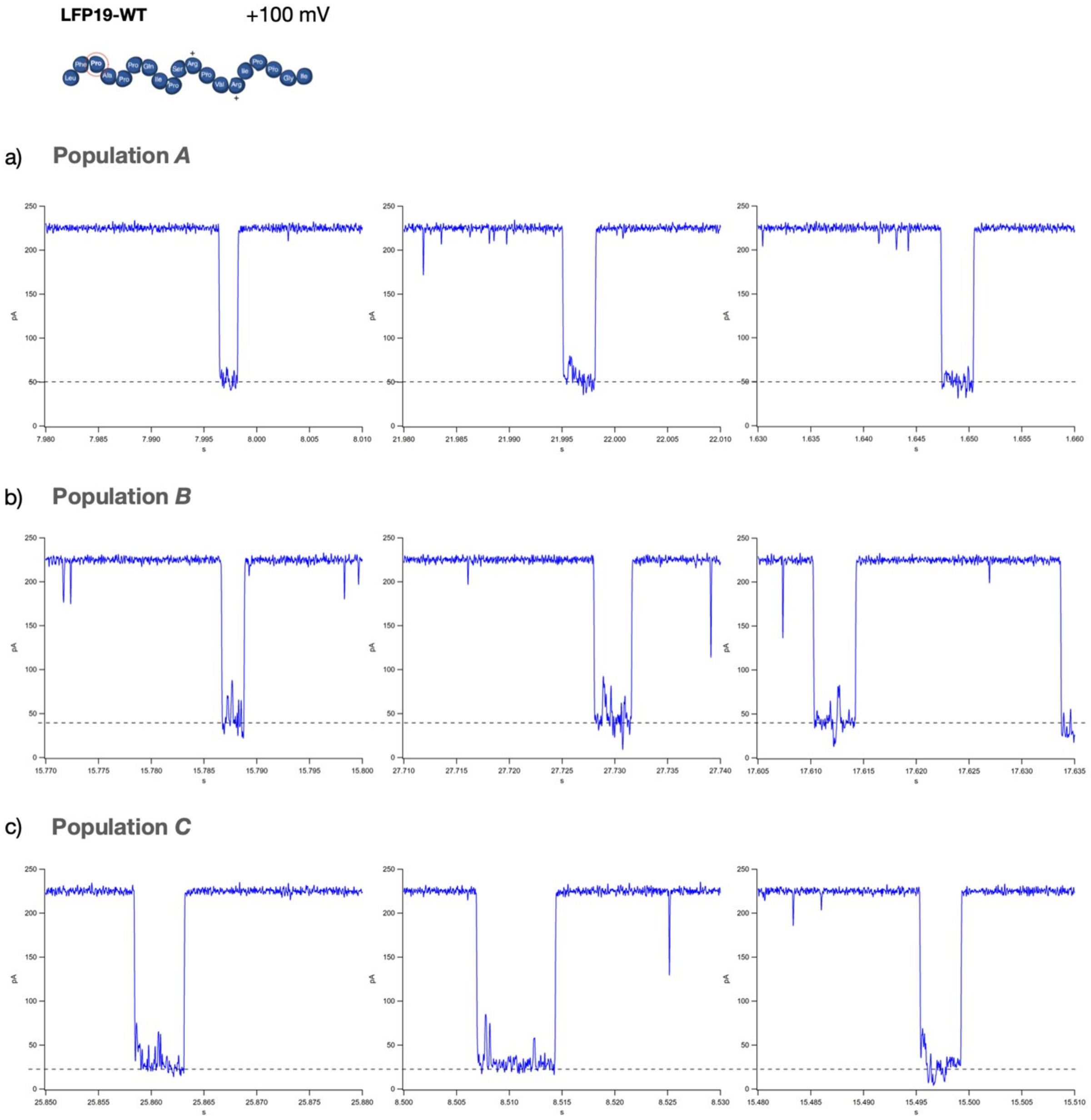

Figure S6. Randomly picked up events of LFP19-WT. Under +100 mV in 4M KCl, a) examples for population *A*; b) examples for population *B*; c) examples for population *C*. Each plot has the same timescale of 30ms.

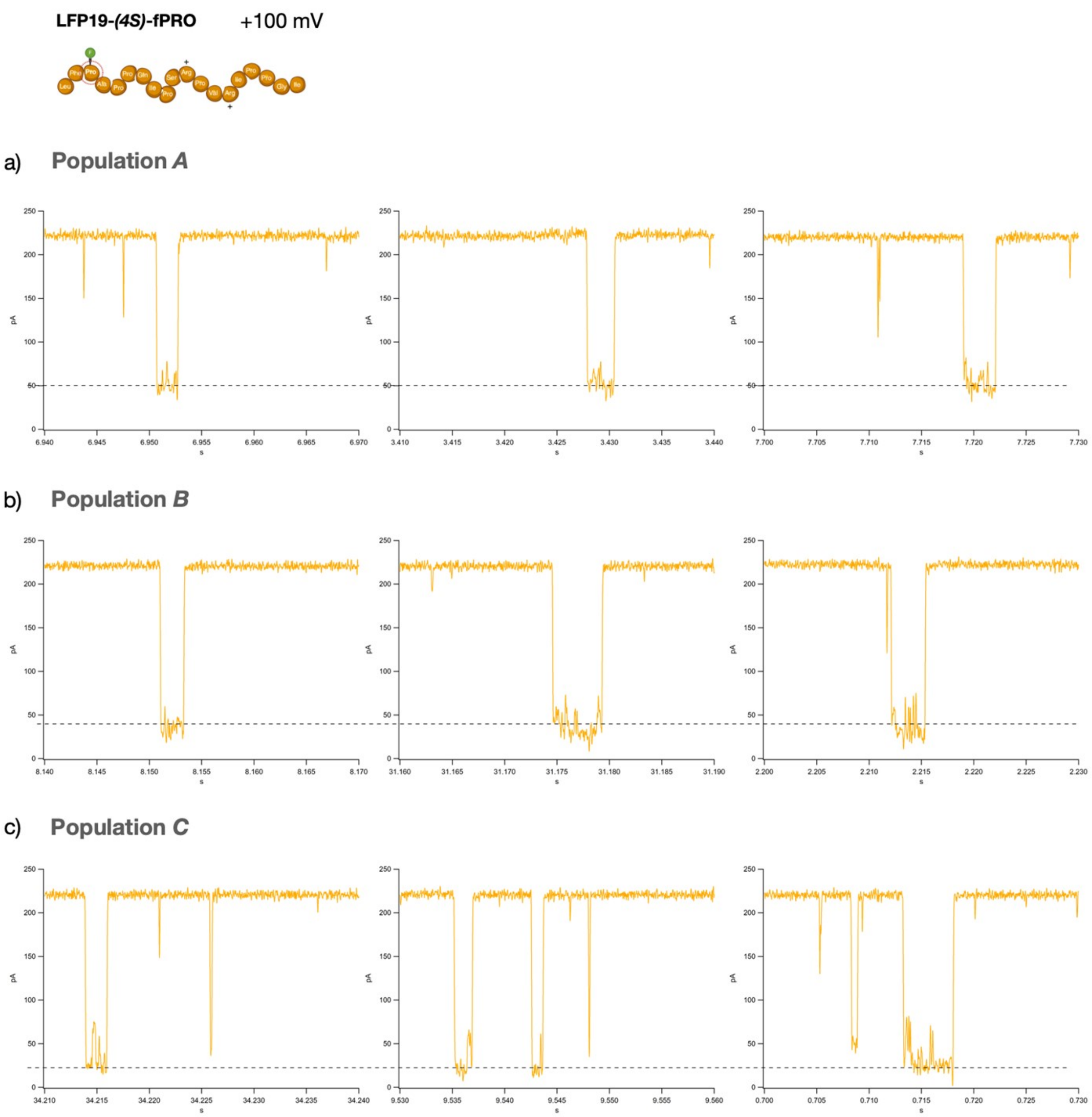

Figure S7. Randomly picked up events of LFP19-*(4S)*-fPRO. Under +100 mV in 4M KCl, a) examples for population *A*; b) examples for population *B*; c) examples for population *C*. Each plot has the same timescale of 30ms.

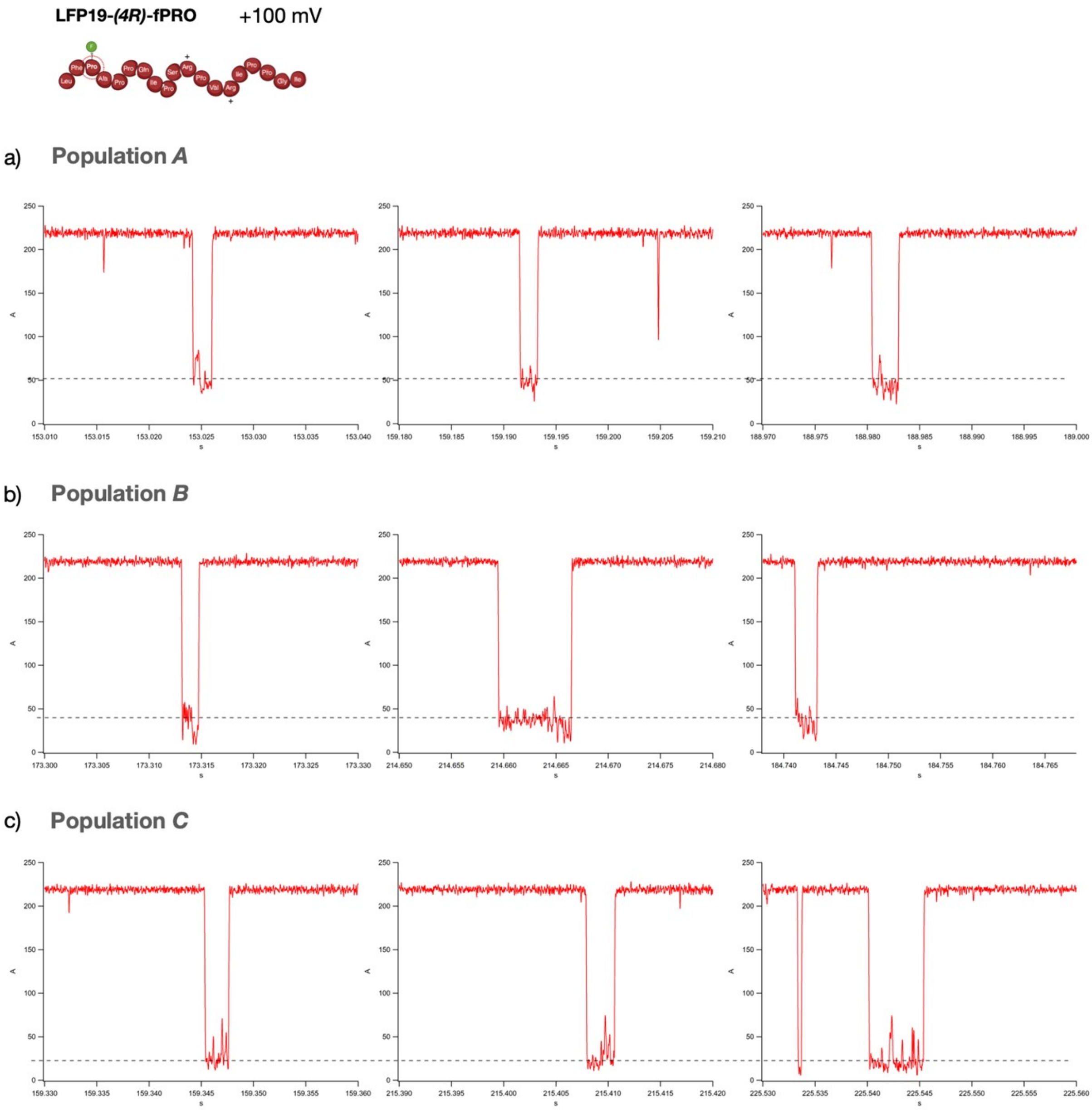

Figure S8. Randomly picked up events of LFP19-*(4R)*-fPRO. Under +100 mV in 4M KCl, a) examples for population *A*; b) examples for population *B*; c) examples for population *C*. Each plot has the same timescale of 30ms.

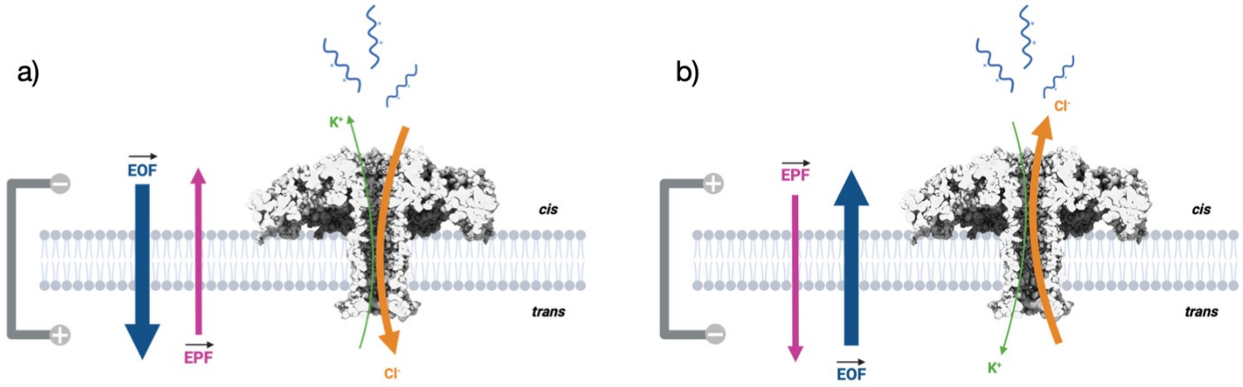

Figure S9. Direction of the driving forces. The positively charged peptides are added on the *cis* side of a two compartments system filled with KCl. Direction under a) positive voltage differential, and b) negative voltage differential. The anion selective Aerolysin is embedded in the bilayer.

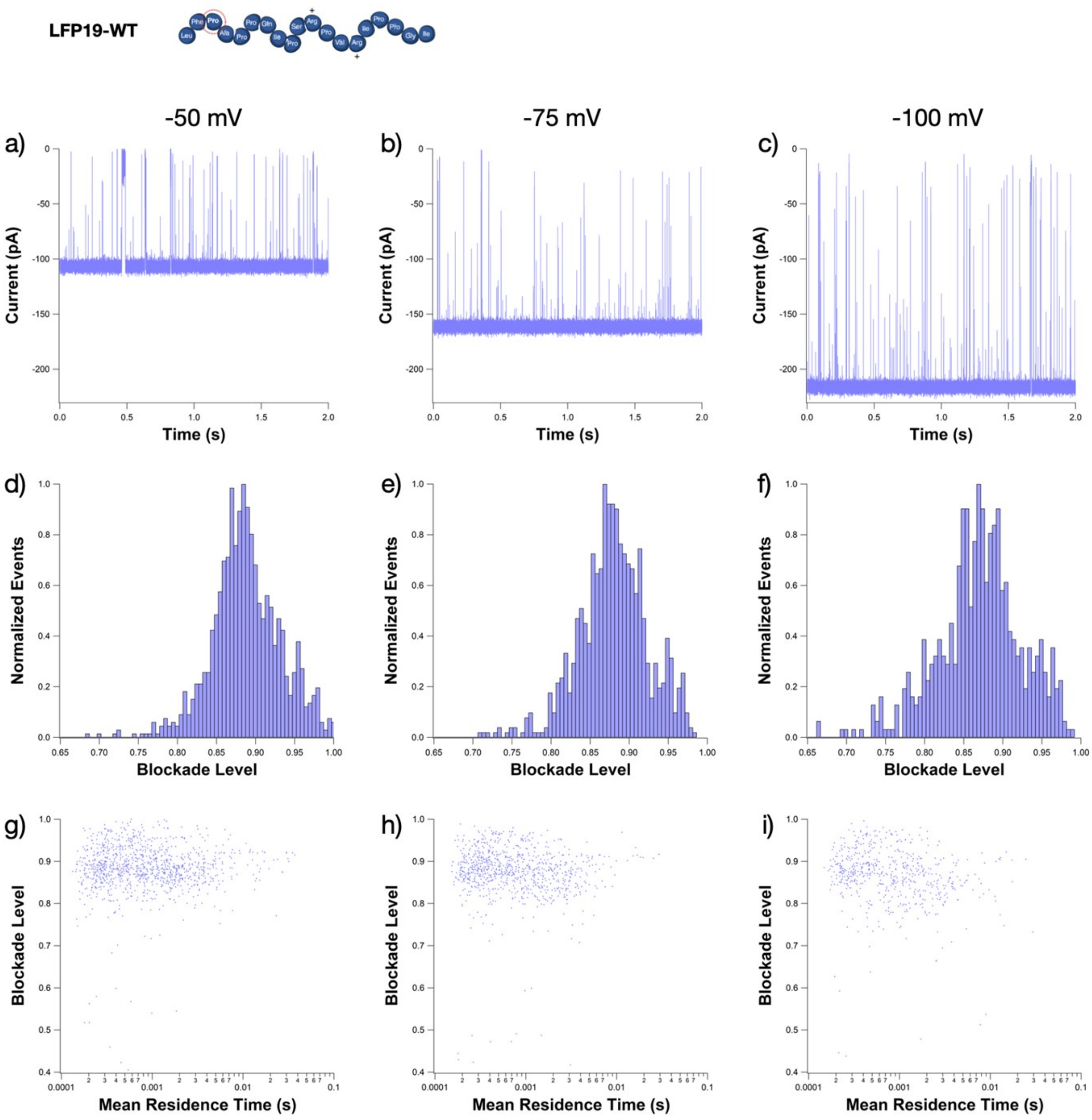

Figure S10. Peptide LFP19-WT under negative voltages in 4M KCl. (a, b, c) current traces for a timescale of 2s. (d, e, f) histograms of the normalized number of events against the normalized average blockade level (ΔI_b). (g, h, i) scatter plot of the normalized average blockade level (ΔI_b) against the mean residence time.

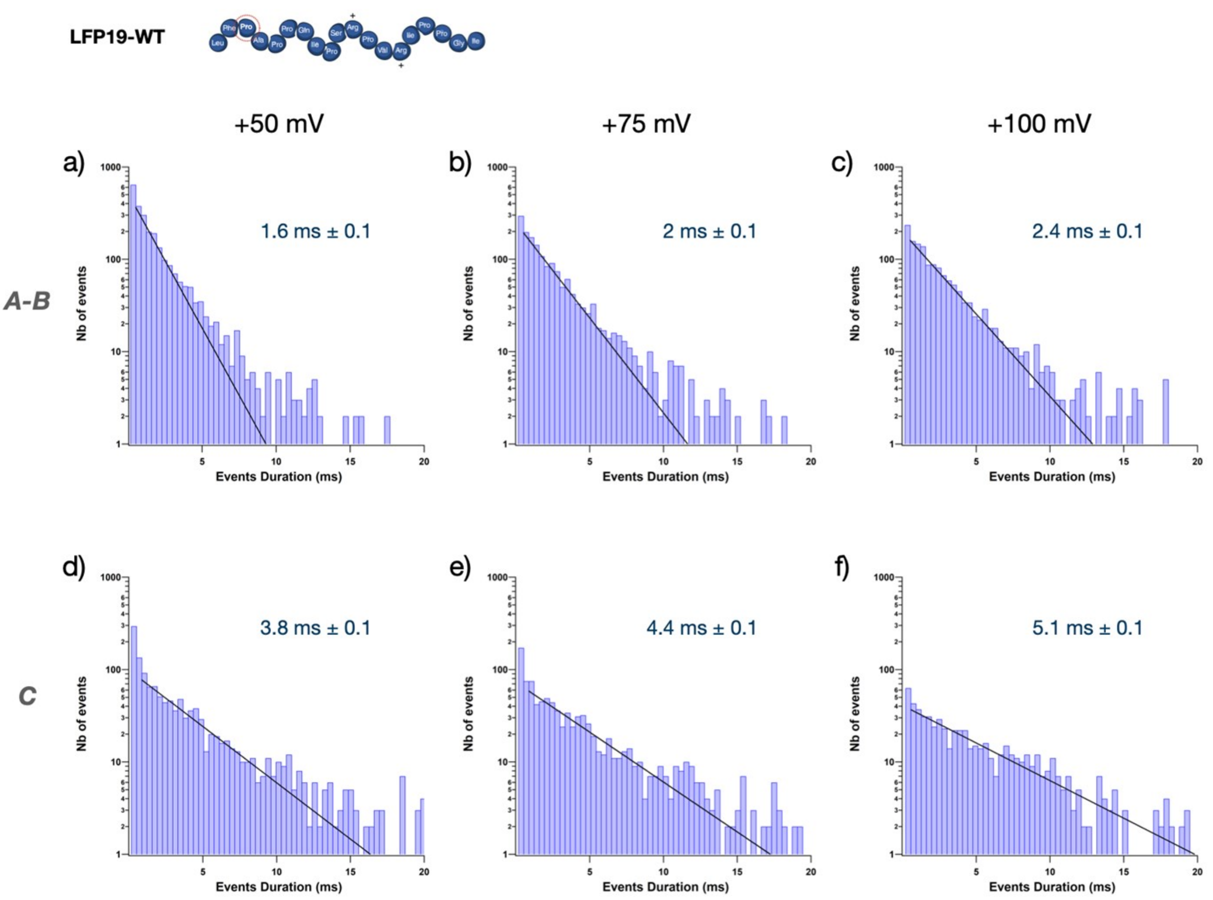

Figure S11. Fittings used to determine the mean residence time for LFP19-WT. (a, b, c) histograms of the number of events for the overlapping populations *A*-*B* against the events duration. (d, e, f) histograms of the number of events for the population *C* against the event duration.

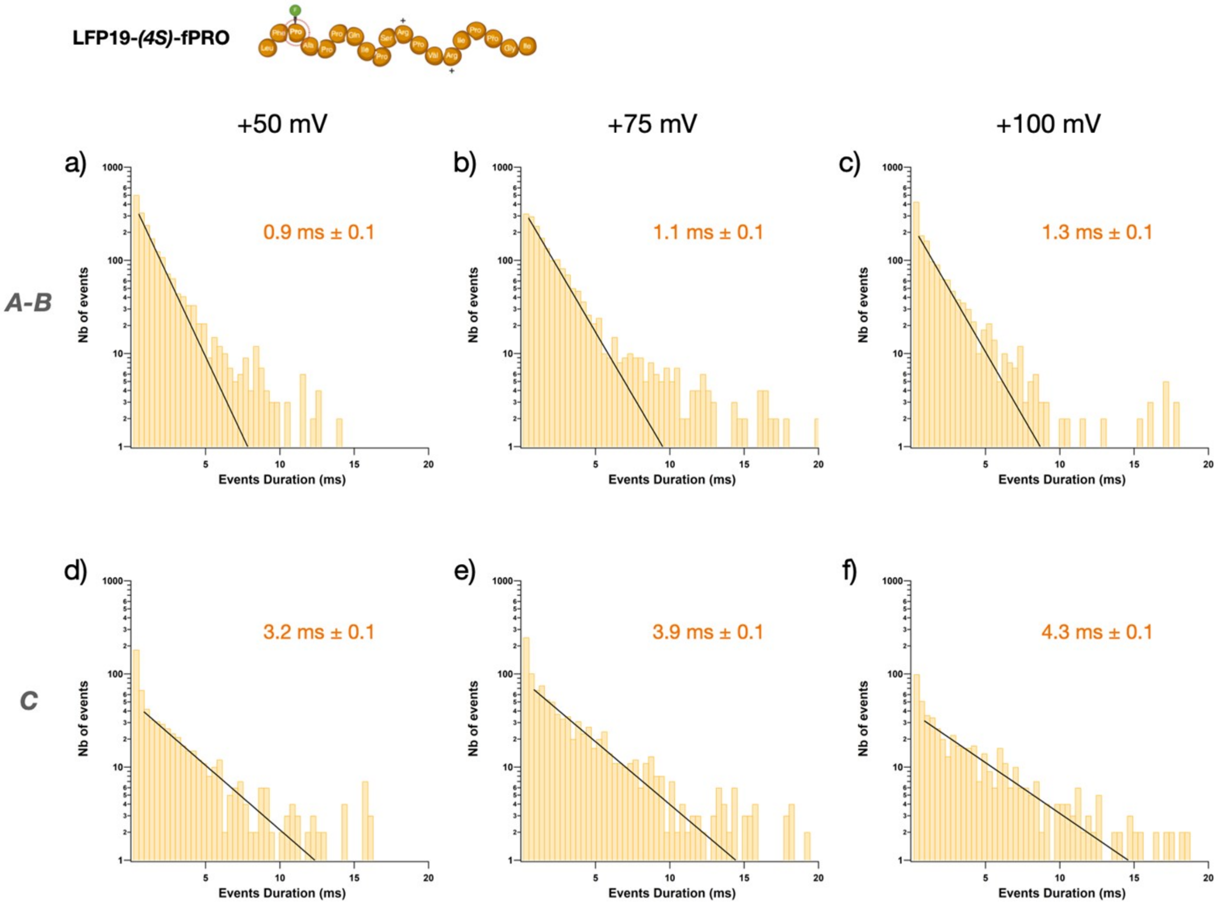

Figure S12. Fittings used to determine the mean residence time for LFP19-*(4S)*-fPRO. (a, b, c) histograms of the number of events for the overlapping populations *A*-*B* against the events duration. (d, e, f) histograms of the number of events for the population *C* against the event duration.

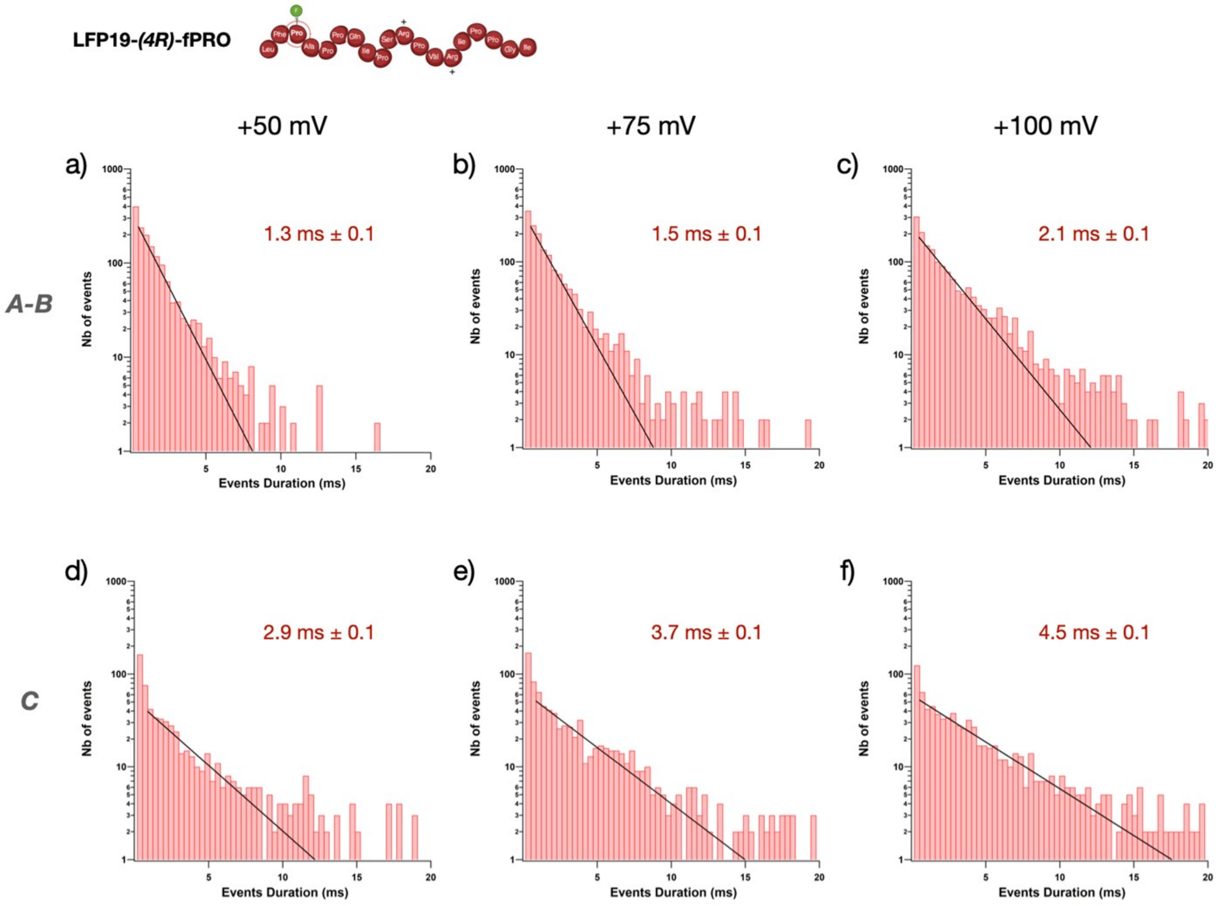

Figure S13. Fittings used to determine the mean residence time for LFP19-*(4R)*-fPRO. (a, b, c) histograms of the number of events for the overlapping populations *A*-*B* against the events duration. (d, e, f) histograms of the number of events for the population *C* against the event duration.

Voltage	KCl [4]M, Tris [25]mM; pH 7.5	CsCl [4]M, Tris [25]mM; pH 7.5
**50 mV**	**25 ± 0.3 Hz**	**4.1 ± 0.1 Hz**
**60 mV**	**26.2 ± 0.8 Hz**	**4.7 ± 0.1 Hz**
**75 mV**	**27.2 ± 0.1 Hz**	**5.4 ± 0.1 Hz**
**100 mV**	**27.7 ± 0.3 Hz**	**6.1 ± 0.1 Hz**
**110 mV**	**28.4 ± 0.6 Hz**	**7.1 ± 0.1 Hz**

Table S1. Global frequency values of 15 µM LFP19-*(4S)*-fPRO 4M KCl and 4M CsCl.

		Exp.1	Ratio Exp. 1			Exp. 2	Ratio Exp. 2			Average		
Voltage		Height B	*trans*	*cis*		Height B	*trans*	*cis*		*trans*	*cis*	STDEV
50 mV		0.54	0.65	0.35		0.54	0.65	0.35		0.65	0.35	0.01
60 mV		0.49	0.67	0.33		0.51	0.66	0.34		0.67	0.33	0.01
75 mV		0.49	0.67	0.33		0.56	0.64	0.36		0.66	0.34	0.02
90 mV		0.56	0.64	0.36		0.44	0.69	0.31		0.67	0.33	0.04
100 mV		0.56	0.64	0.36		0.58	0.63	0.37		0.64	0.36	0.01
110 mV		0.55	0.65	0.35		0.52	0.66	0.34		0.65	0.35	0.01
Total Average	*trans*	*cis*	STDEV							**WT-Pro**		
	0.65	0.35	0.01									
Voltage		Height B	*trans*	*cis*		Height B	*trans*	*cis*		*trans*	*cis*	STDEV
50 mV		0.65	0.61	0.39		0.65	0.61	0.39		0.61	0.39	0.01
60 mV		0.66	0.60	0.40		0.65	0.61	0.39		0.60	0.40	0.01
75 mV		0.68	0.60	0.40		0.6	0.63	0.37		0.61	0.39	0.02
90 mV		0.63	0.61	0.39		0.6	0.63	0.37		0.62	0.38	0.01
100 mV		0.64	0.61	0.39		0.65	0.61	0.29		0.61	0.39	0.01
110 mV		0.61	0.62	0.38		0.6	0.63	0.37		0.62	0.38	0.01
Total Average	*trans*	*cis*	STDEV							***(4S)*fPro**		
	0.61	0.39	0.01									
Voltage		Height B	*trans*	*cis*		Height B	*trans*	*cis*		*trans*	*cis*	STDEV
50 mV		0.43	0.70	0.30		0.48	0.68	0.32		0.69	0.31	0.02
60 mV		0.47	0.68	0.32		0.45	0.69	0.31		0.68	0.32	0.01
75 mV		0.46	0.68	0.32		0.45	0.69	0.31		0.69	0.31	0.01
90 mV		0.44	0.69	0.31		0.4	0.71	0.29		0.70	0.30	0.01
100 mV		0.41	0.71	0.29		0.43	0.70	0.30		0.70	0.30	0.01
110 mV		0.39	0.72	0.28		0.34	0.75	0.25		0.73	0.27	0.02
Total Average	*trans*	*cis*	STDEV							***(4R)*fPro**		
	0.70	0.30	0.02									

Table S2. Values of single voltage average and the total average of *trans/cis* ratio. Events under +50 mV: exp.1 $n_{\text{LFP19-WT}}$ = 2733, $n_{\text{LFP19-}(4S)\text{-fPRO}}$ = 3107, $n_{\text{LFP19-}(4R)\text{-fPRO}}$ = 3145; exp.2 $n_{\text{LFP19-WT}}$ = 5145, $n_{\text{LFP19-}(4S)\text{-fPRO}}$ = 1886, $n_{\text{LFP19-}(4R)\text{-fPRO}}$ = 2043. Events under +60 mV: exp.1 $n_{\text{LFP19-WT}}$ = 5306, $n_{\text{LFP19-}(4S)\text{-fPRO}}$ = 3269,$n_{\text{LFP19-}(4R)\text{-fPRO}}$ = 2184 ; exp.2 $n_{\text{LFP19-WT}}$ = 2681, $n_{\text{LFP19-}(4S)\text{-fPRO}}$ = 2475, $n_{\text{LFP19-}(4R)\text{-fPRO}}$ = 3149. Events under +75 mV: exp.1 $n_{\text{LFP19-WT}}$ = 2753, $n_{\text{LFP19-}(4S)\text{-fPRO}}$ = 2651, $n_{\text{LFP19-}(4R)\text{-fPRO}}$ = 3454; exp.2 $n_{\text{LFP19-WT}}$ = 3599, $n_{\text{LFP19-}(4S)\text{-fPRO}}$ = 3721, $n_{\text{LFP19-}(4R)\text{-fPRO}}$ = 2768. Events under +90 mV: exp.1

$n_{LFP19\text{-}WT}$ = 5190, $n_{LFP19\text{-}(4S)\text{-}fPRO}$ = 3582, $n_{LFP19\text{-}(4R)\text{-}fPRO}$ = 3680; exp.2 $n_{LFP19\text{-}WT}$ = 3653, $n_{LFP19\text{-}(4S)\text{-}fPRO}$ = 3265, $n_{LFP19\text{-}(4R)\text{-}fPRO}$ = 2854. Events under +100 mV: exp.1 $n_{LFP19\text{-}WT}$ = 3391, $n_{LFP19\text{-}(4S)\text{-}fPRO}$ = 2724, $n_{LFP19\text{-}(4R)\text{-}fPRO}$ = 3386; exp.2 $n_{LFP19\text{-}WT}$ = 2545, $n_{LFP19\text{-}(4S)\text{-}fPRO}$ = 2532, $n_{LFP19\text{-}(4R)\text{-}fPRO}$ = 4547. Events under +110 mV: exp.1 $n_{LFP19\text{-}WT}$ = 1855, $n_{LFP19\text{-}(4S)\text{-}fPRO}$ = 1003, $n_{LFP19\text{-}(4R)\text{-}fPRO}$ = 4147; exp.2 $n_{LFP19\text{-}WT}$ = 1997, $n_{LFP19\text{-}(4S)\text{-}fPRO}$ = 3737, $n_{LFP19\text{-}(4R)\text{-}fPRO}$ = 3608. *Exp = experiment; *a* = height value for population *A* from the histogram (Fig 2 and Fig S2); *b* = height value for population *B* from the histogram (Fig 2 and Fig S2).
